# Computed tomography versus ultrasound/fine needle aspiration biopsy in differential diagnosis of thyroid nodules: a retrospective analysis

**DOI:** 10.1016/j.bjorl.2019.10.003

**Published:** 2019-11-17

**Authors:** Wang Tao, Zhu Qingjun, Zheng Wei, Zhou Fang, Zhou Lei, Ni Yuanyuan, Hu Kefu

**Affiliations:** a Gong’an County People’s Hospital, Department of Ultrasound, Gong’an County, Hubei Province, China; b The People’s Hospital of Jinshi, Department of Ultrasound, Jinshi, Hunan Province, China; c Gong’an County People’s Hospital, Department of Medical Cosmetology, Gong’an County, Hubei Province, China; d Gong’an County People’s Hospital, Department of Emergency, Gong’an County, Hubei Province, China; e Gong’an County People’s Hospital, Department of Medical Administration, Gong’an County, Hubei Province, China

**Keywords:** Computed tomography, Fine needle aspiration biopsy, Parenchymatous disease, Thyroid nodule, Ultrasound

## Abstract

**Introduction:**

Ultrasound sonography provides a quick method for determining which nodule to sample for fine needle aspiration biopsy in thyroid nodules. On the other hand, the computed tomography examination is not restricted by echo attenuation and distinguishes between benign and malignant nodules.

**Objective:**

To compare computed tomography examinations against ultrasound/fine needle aspiration biopsy in the differential diagnosis of thyroid nodules.

**Methods:**

Data regarding computed tomography examinations, sonographic finding following fine needle aspiration biopsy, and tumor histology of 953 nodules from 698 patients who underwent thyroidectomy were collected and analyzed. The beneficial score for detection of the malignant tumor for each adopted modality was evaluated.

**Results:**

Ultrasound images did not show a well-circumscribed solid mass in 89 nodules, and ultimately did not detect nodules in fine needle aspiration biopsies (false positive non-malignant nodules). Ultrasound images showed parenchymatous disease (false positive malignant nodules) in several nodules. Computed tomography examinations demonstrated higher difficulty in detection of malignant nodules of 1.0–2.0 cm size than ultrasound examination following fine needle aspiration biopsies; compared to tumor histological data, computed tomography examinations had a sensitivity of 0.879.

**Conclusion:**

Computed tomography examinations are a more reliable method for differential diagnosis of thyroid nodules than ultrasound examinations followed by fine needle aspiration biopsy.

**Level of Evidence:**

III.

## Introduction

In Chinese women, thyroid cancer is the most diagnosed cancer before the age of 30 years and has a stable mortality rate.^1^ If patients with normal life expectancy have had focal metabolic activity in the thyroid gland, then thyroid ultrasound is recommended.^2^ Nowadays, the incidence of thyroid malignancy has increased, but thyroid nodules are mostly benign.^3^ Fine needle aspiration biopsy, ultrasound examinations of the neck, Computed Tomography (CT), Magnetic Resonance Imaging (MRI), and ^18^F-fluor-Deoxyglucose Positron Emission Tomography (FDG PET) are used regularly for screening thyroid nodules.^4^ In such situations, accurate diagnosis by all diagnostic methods is crucial.

The ultrasound sonography can increase diagnostic accuracy for thyroid malignancy and provides a quick method for determining which nodule to sample for fine needle aspiration biopsy. Also, sonography prevents surgical procedures and unnecessary invasive tissue sampling.^5^ In ultrasound, the presence of suspicious features, for example, microcalcifications, irregular margins, marked hypoechogenicity, or taller than the wide shape is considered as thyroid nodules.^6^ These suspicious features of ultrasound have good interobserver agreements.^7^ Thyroid nodules are often detected on chest CT examinations.^4^ Unlike ultrasound, CT examination is not restricted by echo attenuation and can fully show the size and shape of the nodules. Also, CT distinguishes between benign and malignant nodules^8^ but CT has limitations in the differentiation of malignant and non-malignant nodules.^4^ Nodule size is a more reliable parameter for malignancy prediction.^9^ A retrospective study is given a predictive model for malignancy using patients’ age, nodule size, and fine needle aspiration biopsy^10^ but a retrospective cohort analyses showed that increasing the risk of cancer is associated with nodule size in a nonlinear fashion.^4^, ^9^, ^11^, ^12^ A prospective study suggests thyroid lobectomy for nodules of 4 cm or more.^13^ While some surgeons are recommending surgical resection without fine needle aspiration biopsy for nodules ≥ 4 cm because the reliability of fine needle aspiration biopsy is not influenced by the size of the nodule.^14^ In addition to this, in a retrospective study, a significant discrepancy is reported between sonographic measurements and tumor histological diameter for nodules greater than 1.5 cm.^15^ All in all, there is a dilemma about the differential diagnosis of thyroid nodules among sonographic examinations, fine needle aspiration biopsy findings, and CT examinations.

The objective of the study was to compare CT examinations against ultrasound following fine needle aspiration biopsy in the differential diagnosis of thyroid nodules considering tumor histological data as ‘gold standard’ in Chinese patients who underwent thyroidectomy at level III of evidence.

## Methods

### Ethics consideration and consent to participate

The protocol (GCP/CL/29/19 dated 27 January 2019) had been approved by the Gong’an County People’s Hospital review board. The study had adhered to the law of China, the Declarations of Helsinki version 2008, and the Strengthening the Reporting of Observational studies in Epidemiology (STROBE) statement. An informed consent form had been signed by all patients regarding radiology, biopsies, anesthesia, surgeries, pathology, and publications of the study in all formats of the publication house including personal data of patients irrespective of time and language. Being a retrospective study, the clinical trial registration had been waived by the institutional review board.

### Inclusion criteria

The medical records of patients who underwent partial or total thyroidectomy were reviewed.

### Exclusion criteria

Patients who had incomplete records and age less than 18 years were excluded from the analysis. Nodules in which definitive correlations were not established were excluded from the analysis.

### Data collection

Medical records of patients were reviewed and the demographic and clinical characteristics of patients were studied. Data regarding CT examinations, sonographic finding following fine needle aspiration biopsy (according to the Bethesda system for reporting thyroid cytopathology), and tumor histology were collected. If patients had multiple nodules then each lesion was considered separately.

### Decision curve analysis

The beneficial score for detection of the malignant tumor for each adopted modality was evaluated as per Eq. 1 and 2:^16^ The risk of overdiagnosis was considered as per American Thyroid Association (ATA) guidelines that less than 1 cm thyroid nodules do not need further investigation.^4^

(1) $$\text{Beneficial}\;\text{score}=\frac{\text{Numbers}\;\text{of}\;\text{true}\;\text{positive}\;\text{malignant}\;\text{nodules}}{\text{Total}\;\text{numbers}\;\text{of}\;\text{nodules}\;\text{screened}}-\left[\frac{\text{Numbers}\;\text{of}\;\text{false}\;\text{positive}\;\text{malignant}\;\text{nodules}}{\text{Total}\;\text{numbers}\;\text{of}\;\text{nodules}\;\text{screened}}\;\times \text{Risk}\;\text{of}\;\text{overdiagnosis}\right]$$

(2) $$\text{Risk}\;\text{of}\;\text{overdiagnosis}=\frac{7}{\text{Nodule}\;\text{size}-1}$$

### Statistical analysis

InStat (version window, GraphPad Software, San Diego, CA, USA) was used for statistical analysis. Categorial data were analyzed by the Chi-Square test or the Fischer exact test. Continuous data were analyzed by one-way analysis of variance (ANOVA) following Tukey-Kramer Multiple Comparisons Test (considering critical value [*q*] > 3.314 as significant).^15^ All results were considered significant at 95 % of confidence level.

## Results

### Participants

**Fig. 1:**
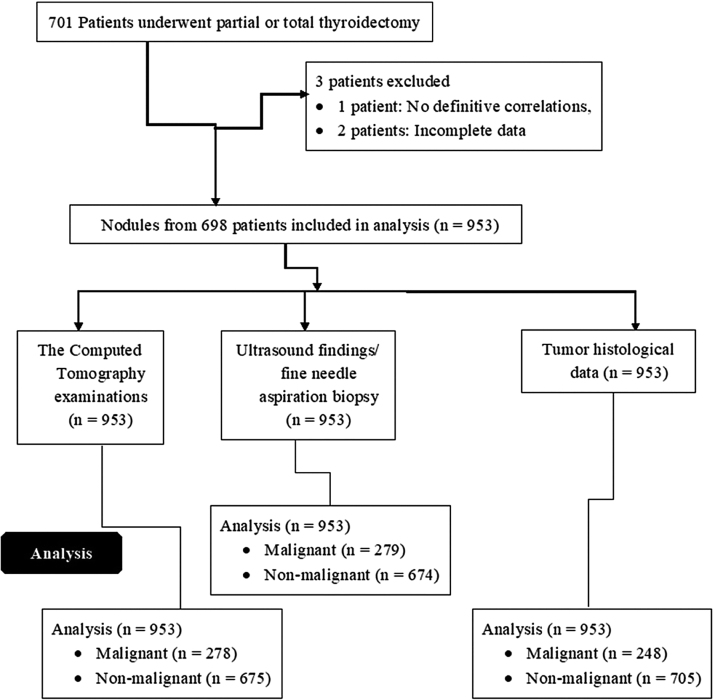
Flow diagram of the analysis.

A total of 701 patients underwent partial or total thyroidectomy from 1 January 2017 to 15 January 2019 in the Gong’an County People’s Hospital, China and the People’s Hospital of Jinshi City, China. Among them, definitive correlations were not established for one patient and two patients had incomplete data available in the medical records. Therefore, they were excluded from the study. Data regarding CT examinations and sonographic finding following fine needle aspiration biopsies, and tumor histology of 953 nodules from 698 patients were included in the analysis (Fig. 1).

### Characteristics of study participants

Out of 698 patients, 482 patients had a single nodule, 183 patients had two nodules, 30 patients had three nodules, one patient had four nodules, one patient had five nodules, and one patient had six nodules. There were significant higher nodule sizes detected by sonographic finding (*p* < 0.0001, *q* = 17.57) and CT examinations (*p* < 0.0001, *q* = 7.53) than tumor histological data. The other demographical and clinical parameters of the patients who underwent thyroidectomy are presented in Table 1.

**Table 1:** Demographic and clinical characteristics of the enrolled patients.

Characteristics		Parameters
Patients included in the analysis		698
Nodules included in the analysis		953
Age (years)	Minimum	18
	Maximum	65
	Mean ± SD	49.81 ± 9.45
Gender	Female	497 (71)
	Male	201 (29)
Body Mass Index (kg/m^2^)		24.52 ± 2.55
Family history	Yes	283 (41)
	No	415 (59)
Numbers of nodules	1	482 (69)
	2	183 (26)
	3	30 (4.4)
	4	1 (0.2)
	5	1 (0.2)
	6	1 (0.2)
Nodule size by sonographic finding (cm)	Minimum	0.5
	Maximum	7.0
	Mean ± SD	2.45 ± 1.18
Nodule size by computed tomography (cm)	Minimum	0.1
	Maximum	6.7
	Mean ± SD	1.89 ± 0.95
Nodule size by tumor histological data (cm)	Minimum	0.08
	Maximum	6.53
	Mean ± SD	1.65 ± 0.78

Constant variables are presented as frequency (percentage) and continuous variables are presented as mean ± SD.

### Size and characteristics of nodules

According to ultrasound findings followed by fine needle aspiration biopsy, a total of 279 nodules were malignant and 674 nodules were non-malignant (Table 2).

**Table 2:** Size and characteristics of nodules according to ultrasound findings followed by fine needle aspiration biopsy.

Size (cm)	Total nodule	Characteristics of nodules	
		Malignant nodules	Non-malignant tumors
Numbers of nodules reviewed	953 (100)	279 (29)	674 (71)
≤0.99	267 (28)	35 (3)	232 (25)
1.0–1.99	346 (36)	172 (18)	174 (18)
2.0–2.99	145 (15)	45 (5)	100 (10)
3.0–3.99	95 (10)	12 (1)	83 (9)
4.0–4.99	60 (6)	8 (1)	52 (5)
5.0–5.99	25 (3)	4 (0.5)	21 (2.5)
≥ 6	15 (2)	3 (0.5)	12 (1.5)

Variables are presented as frequency (percentage).

Presence of calcification, hypoechoic nodule, blurred margins, intramodular vascular pattern, or heterogeneous echogenicity mass was considered as malignancy.

According to CT examinations, a total of 278 nodules were malignant and 675 nodules were non-malignant (Table 3).

**Table 3:** Size and characteristics of nodules according to the Computed Tomography examinations.

Size (cm)	Total nodule	Characteristics of nodules	
		Malignant nodules	Non-malignant tumors
Numbers of nodules reviewed	953 (100)	278 (29)	675 (71)
≤ 0.99	291	12 (1.2)	279 (29)
1.0–1.99	275	189 (19.8)	86 (9)
2.0–2.99	160	57 (6)	103 (11)
3.0–3.99	110	7 (0.7)	103 (11)
4.0–4.99	69	5 (0.5)	64 (7)
5.0–5.99	24	7 (0.7)	17 (2)
≥ 6	13	1 (0.1)	12 (1)

Variables are presented as frequency (percentage).

The anteroposterior dimension to the transverse dimension ratio > 1.0 or means attenuation > 130 HU was considered as malignancy.

According to tumor histological data after thyroidectomy, 248 nodules were malignant and 705 nodules were non-malignant. Among the malignant tumors, 219 were papillary thyroid carcinomas, 17 were follicular thyroid carcinomas, six were poorly differentiated carcinoma, three were anaplastic thyroid carcinoma, one was medullary thyroid carcinoma, and two were metastatic carcinoma (Table 4).

**Table 4:** Size and characteristics of nodules according to tumor histological data.

Size (cm)	Characteristics of nodules							
	Malignant nodules							Non-malignant tumors
	Papillary thyroid carcinoma	Follicular thyroid carcinoma	Poorly differentiated carcinoma	Anaplastic thyroid carcinoma	Medullary thyroid carcinoma	Metastatic carcinoma	Total	
≤ 0.99	45 (5)	1 (0.1)	0 (0)	1 (0.1)	1 (0.1)	1 (0.1)	49 (5.4)	250 (26)
1.0–1.99	86 (9)	2 (0.2)	5 (0.5)	1 (0.1)	0 (0)	1 (0.1)	95 (9.9)	201 (21)
2.0–2.99	41 (4)	8 (1)	1 (0.1)	0 (0)	0 (0)	0 (0)	50 (5.1)	101 (10)
3.0–3.99	24 (3)	3 (0.3)	0 (0)	0 (0)	0 (0)	0 (0)	27 (3.3)	80 (8.4)
4.0–4.99	13 (1)	2 (0.2)	0 (0)	1 (0.3)	0 (0)	0 (0)	16 (1.5)	48 (5)
5.0–5.99	7 (1)	1 (0.1)	0 (0)	0 (0)	0 (0)	0 (0)	8 (1.1)	15 (2)
≥ 6	3 (0.3)	0 (0)	0 (0)	0 (0)	0 (0)	0 (0)	3 (0.3)	10 (1)
Total	219 (23.3)	17 (1.9)	6 (0.6)	3 (0.5)	1 (0.1)	2 (0.2)	248 (26.6)	705 (73.4)

Variables are presented as frequency (percentage).

According to the Bethesda system for reporting thyroid cytopathology.

Among the malignant nodules, the highest numbers of nodules were predicted in 1.0–1.99 cm size, then after 2.0–2.99 cm followed by less than 0.99 cm nodule size.

### Diagnostic parameters

Ultrasound findings/fine needle aspiration biopsy had the same true positive malignant nodules as that detected by tumor histological data (*p* = 0.999) (Table 5).

**Table 5:** Diagnostic parameters for adopted modalities.

Nodules	Tumor histological data	Ultrasound findings/fine needle aspiration biopsy		The computed tomography examinations	
Numbers of nodules reviewed	953	953	ap-value	953	ap-value
True positive malignant nodules	248 (26)	247 (26)^b^	0.999	210 (22)	0.047
True positive non-malignant nodules	705 (74)	585 (62)	<0.0001	560 (59)	<0.0001
False positive malignant nodules	0 (0)	32 (3)	<0.0001	68 (7)	<0.0001
False positive non-malignant nodules	0 (0)	89 (9)	<0.0001	115 (12)	<0.0001
Sensitivity	1	0.907	<0.0001	0.879	<0.0001
Specificity	1	0.873	<0.0001	0.808	<0.0001

Variables are presented as frequency (percentage).

The Chi-Square independence test was used for statistical analysis.

A *p* <  0.05 was considered significant.

a With respect to tumor histological data.

b Insignificant difference with respect to tumor histological data.

**Fig. 2:**
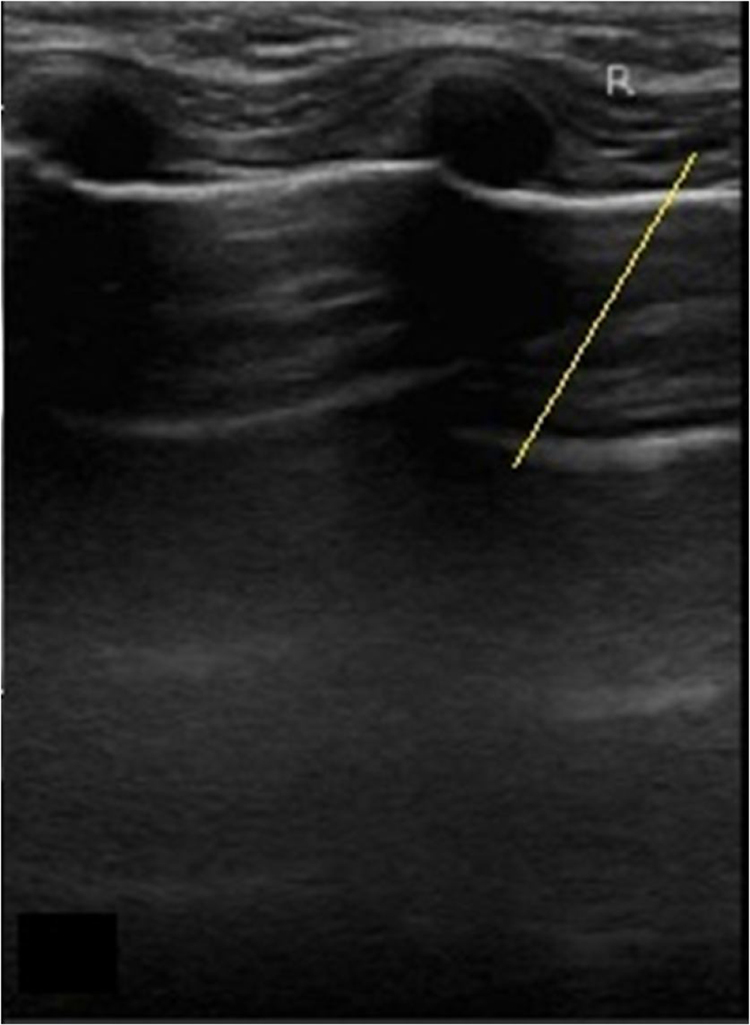
False positive non-malignant nodules by ultrasound. 68 years woman with papillary thyroid carcinoma diagnosed by tumor histological data. Transverse ultrasound image of the right thyroid lobe. Arrow indicates hypoechoic mass.

**Fig. 3:**
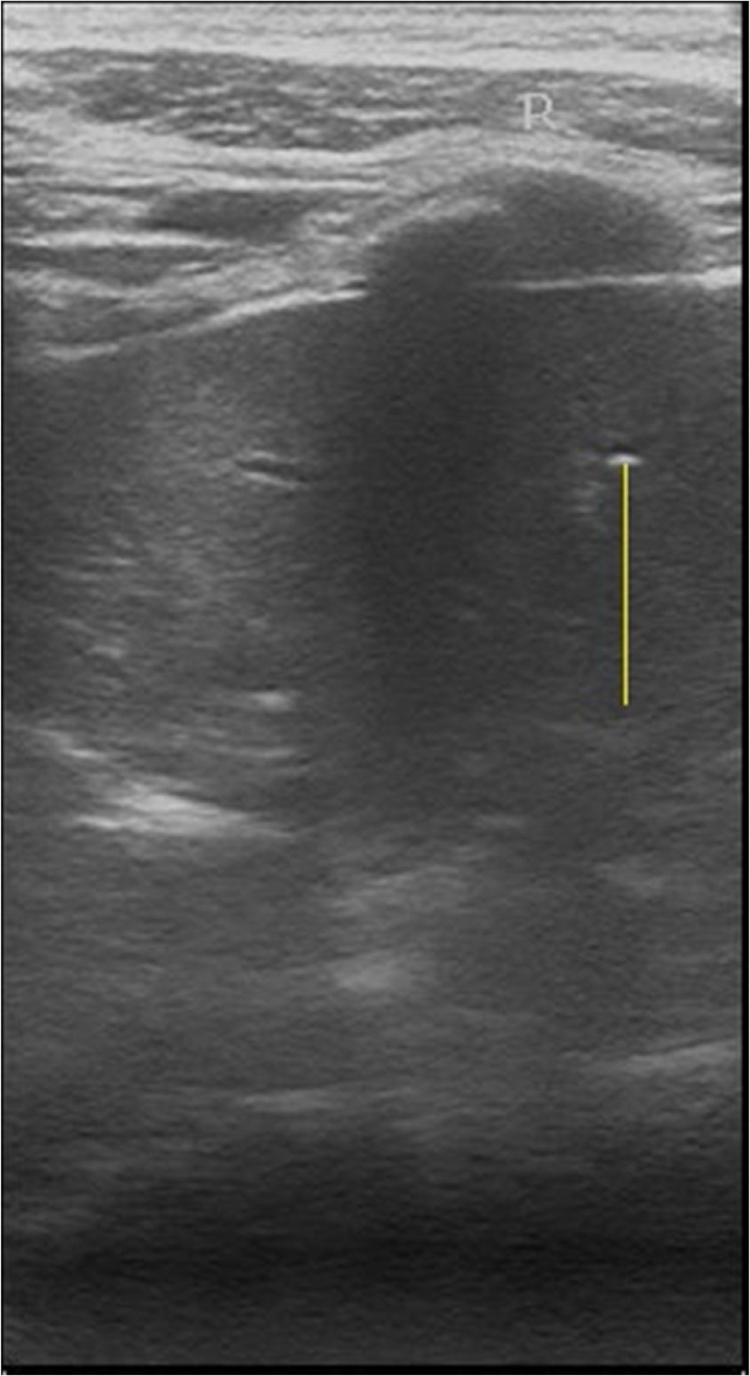
False positive malignant nodules by ultrasound. 53 years woman with non-malignant thyroid tumor diagnosed by tumor histological data. Transverse ultrasound image of the right thyroid lobe. Arrow indicates diffuse parenchymatous disease.

There was no significant difference for detected numbers of malignant and non- malignant nodules between CT examinations and tumor histological data (*p* = 0.137) and between ultrasound findings followed by fine needle aspiration biopsy and tumor histological data (*p* = 0.124), but ultrasound images did not show a well-circumscribed solid mass in 89 nodules, and ultimately did not detect malignancyin fine needle aspiration biopsies (false positive non-malignant nodules) (Fig. 2). Ultrasound images showed parenchymatous disease (false positive malignant nodules) in several nodules (Fig. 3), which were detected as non-malignant nodules in fine needle aspiration biopsies. Still, ultrasound following fine needle aspiration biopsies had 32 false positive malignant nodules as compared to tumor histological data.

**Fig. 4:**
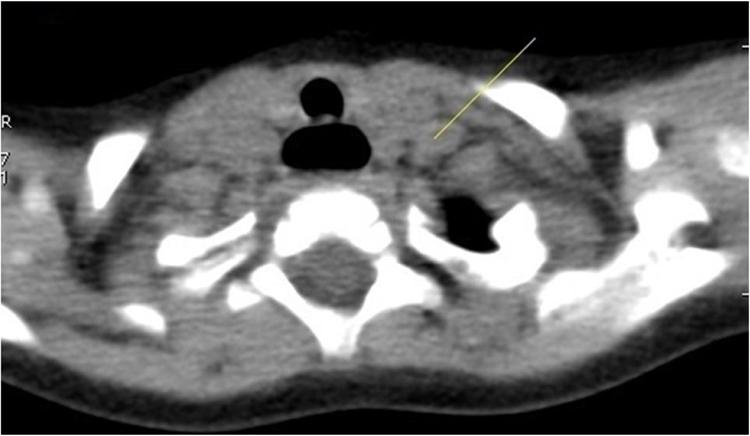
False positive non-malignant nodules by the Computed Tomography. 52 years woman with papillary thyroid carcinoma diagnosed by tumor histological data. The Computed Tomography image of the right thyroid lobe. Arrow indicates low-density mass.

**Fig. 5:**
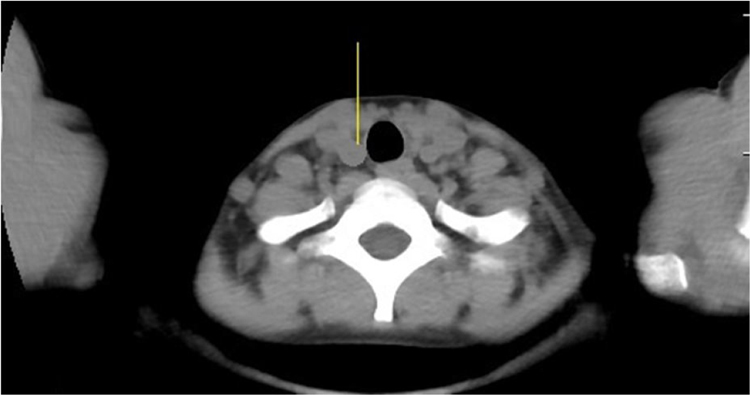
False positive malignant nodules by the Computed Tomography. 49 years woman with non-malignant nodule diagnosed by tumor histological data. The Computed Tomography image of the right thyroid lobe. Arrow indicates well-circumscribed mass.

CT indicates false positive non-malignant nodules in 115 nodules because they had less than 1 the anteroposterior dimension to the transverse dimension ratio (Fig. 4). While in 68 case false positive malignant nodules because of high attenuation value of thyroid nodule were shown (Fig. 5).

### Decision making for thyroidectomy

**Fig. 6:**
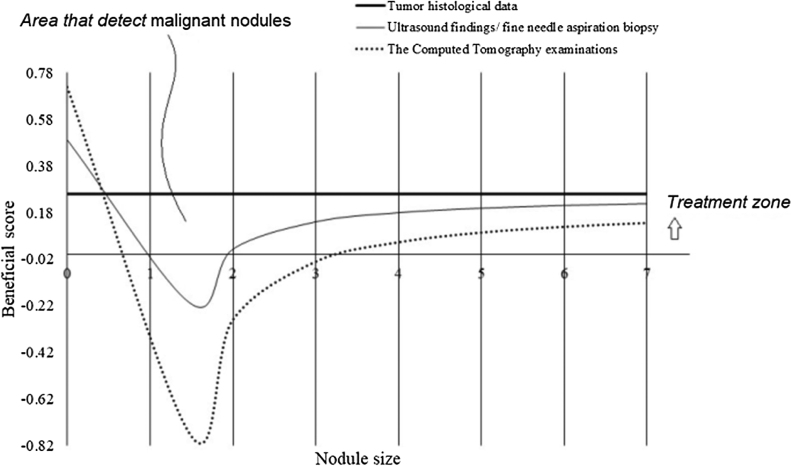
Decision curve analysis for thyroidectomy as per American Thyroid Association guidelines.

For malignant nodules of size range of 1.0–2.0 cm, ultrasound findings/fine needle aspiration biopsy and CT examinations both had a risk of overdiagnosis and overtreatment. Between both CT examinations and ultrasound had high difficulties in detection of malignant nodules of 1.0–2.0 cm size and the beneficial score was also lowest for any sized malignant nodule by CT examinations (Fig. 6).

## Discussion

### Imaging examinations

There were significantly higher nodule sizes for sonographic findings and CT examinations as compared to tumor histological data. Apparent coalescence of nodules on imaging is responsible for showing higher nodule size.^7^ The results of the study were consistent with the retrospective studies.^7^, ^12^, ^15^, ^17^ Sonographic finding and CT examinations apparently overstate the nodulesize compared to pathologic examination.

### Ultrasound/fine-needle aspiration biopsy

During the study of ultrasound following fine-needle aspiration, biopsies were reported of false positive non-malignant nodules. The difference in ultrasound attenuation between the nodule and the surrounding tissue of thyroid is not enough to clearly specify malignant nodules^12^ but in CT mean attenuation > 130 HU was considered as malignancy. The results of the study were consistent with the retrospective studies.^12^, ^18^ Moreover, ultrasound has difficulty in the detection of papillary thyroid carcinoma.^5^ Ultrasound has a restriction for differential diagnosis of thyroid nodules than CT.

During the study, ultrasound examinations showed several malignant nodules which were detected as false positive malignant nodules by fine needle aspiration biopsies. Ultrasound showed diffuse parenchymal disease responsible for false positive malignant nodules.^12^ The results of the study were consistent with the retrospective studies.^12^, ^18^ Fine needle aspiration biopsy is a reliable method to overcome false positive malignant nodules detected by ultrasound images but it is expensive and tedious method.^18^ Also, improper sampling and suboptimal slide preparation in fine needle aspiration biopsy may also be responsible for false positive malignant nodules detection.^14^ In any case, the issue of detection of false positive malignant nodules is difficult to modify by ultrasound examinations.

### CT examinations

There was no significant difference for differential diagnosis of thyroid nodules between CT examinations and tumor histological data (*p* = 0.137). Also, compared to tumor histological data, CT examinations had a sensitivity of 0.879. CT is a reliable objective method for detecting the density of calcification.^8^ The results of the study were consistent with a retrospectively study.^4^ For solitary coarse calcification of uneven density, CT examinations is an easier imaging modality than the other diagnostic methods.

### Limitations

In the limitations of the study, for examples, CT examinations manifested more difficulties in detection of malignant nodules of 1.0–2.0 cm size than ultrasound images. Microcalcifications of thyroid nodules cannot be identified by CT but are identified by ultrasound images clearly.^12^ Also, in the study, significant false positive malignant nodules were also reported by CT examinations (*p* < 0.0001). In ultrasound, central or internal blood flow of the nodule is responsible for malignancy^5^ diagnosis but after intravenous administration of contrast agent solid nodules show increased attenuation (*e.g.* an increase in 1 mg/mL of iodine concentration can increase 26 HU attenuation) or anteroposterior dimension to the transverse dimension ratio,^19^ which shows a non-malignant nodule as malignant one. The results of the study were consistent with the retrospective study.^12^ In the differential diagnosis of thyroid nodules, CT cannot completely replace ultrasound. The work-up was a retrospective study and involves a higher chances of selection bias. The retrospective analysis provided limited information for images than a more dynamic study.

## Conclusion

Computed Tomography is a more reliable method for differential diagnosis of thyroid nodules than ultrasound examinations followed by fine needle aspiration biopsy. A large dynamic study is required to prove the superiority of Computed Tomography over traditional ultrasound examinations in the differential diagnosis of thyroid nodules.

## Availability of data and materials

The datasets used and analyzed during the current study available from the corresponding author on reasonable request.

## Conflicts of interest

The authors declare no conflicts of interest.
